# A bioinformatics analysis revealing autophagy related DEGs in head and neck squamous cell carcinoma: expanding insights into lipid metabolism

**DOI:** 10.1016/j.bjorl.2026.101779

**Published:** 2026-03-10

**Authors:** Yi Cheng, Xiaoyu Li

**Affiliations:** a Jining Medical University, School of Clinical Medicine, Jining, Shandong Province, China; b The Affiliated Hospital of Jining Medical University, Departments of Otolaryngology-Head and Neck Surgery, Jining, China

**Keywords:** Head and neck squamous carcinoma, Autophagy-related genes, Bioinformatics analysis, Prognostic model, Cell migration, GSK3B

## Abstract

**Objective:**

To identify value of prognostic model constructed based on tumor module-associated differential autophagy genes in Head and Neck Squamous Cell Carcinoma (HNSC), and verified through in vitro and tissue experiments.

**Methods:**

After obtaining the relevant information of HNSC patients from the GEO and TCGA databases, we used Weighted Correlation Network Analysis (WGCNA), multivariate Cox regression analysis, and survival analysis to construct a prognostic prediction model, and validated the prognostic value of the model. We used RT-qPCR and cell scratch assays to validate in vitro for the biological results with TU686 and CAL-27 cell lines. We collected pathological sections from 30 patients with laryngeal cancer, hypopharyngeal cancer, and 10 patients with vocal cord polyps, and immunohistochemical DAB staining was performed to detect the expression of GSK3B in these tissues.

**Results:**

A total of 6163 differentially expressed genes were jointly identified between HNSC and control samples. WGCNA analysis revealed that the genes clustered in the brown module had the highest correlation with HNSC. Using the formula derived from the inclusion of 7 genes in the prognostic model, the risk scores were calculated for the training and validation sets of HNSC disease samples. Survival analysis results showed a significant difference in Overall Survival (OS) between the high and low-risk groups in both the training and validation sets (p < 0.05). The one-year, three-year, and five-year AUC values of the risk scores obtained from the prognostic model in the training and validation sets were all greater than 0.6. After knocking down GSK3B, the relative expression levels of si-GSK3B in the si-GSK3B1 and si-GSK3B2 groups were significantly lower than those in the NC group (both p < 0.05). We compared with the NC group, the migration ability of TU686 and CAL-27 cells was significantly decreased (both p < 0.05). We used immunohistochemical DAB method to detect and found that the content of GSK3B in laryngeal cancer was significantly higher than that in vocal cord polyps (p < 0.05), and the content of GSK3B also in pharyngeal cancer was significantly higher than that in vocal cord polyps (p < 0.05).

**Conclusion:**

Prognostic model constructed based on tumor module-associated differential autophagy genes in HNSC holds prognostic value.

**Level of evidence:**

Level 3.

## Highlights

• A successful establishment of a prognostic model for head and neck tumors.
• Differential autophagy genes in tumor models.
• A bioinformatics analysis about head and neck squamous cell carcinoma.

## Introduction

Head and Neck Squamous Cell Carcinoma (HNSCC) is the 6th most common cancer globally. Viral infections, particularly HPV infection, as well as factors such as smoking and alcohol consumption, may contribute to the development of this disease. Treatment options for HNSCC include surgery, radiotherapy, and chemotherapy, which are chosen based on the specific location of the tumor and the stage of the disease. Despite advancements in the diagnosis and treatment of HNSCC, overall survival rates have remained largely unchanged. Autophagy is a process that involves the engulfment of cellular proteins or organelles and their encapsulation in vesicles, which then fuse with lysosomes for degradation of the enclosed contents. Autophagy plays a crucial role in both physiological and pathological processes, with its function varying depending on the integrity of the process, the cellular environment, and the stage of tumor development.^1^ Autophagy helps maintain genomic stability, prevents chronic tissue damage and inflammation, and thereby hinders tumor initiation, proliferation, invasion, and metastasis, particularly in the early stages of cancer.^2^ Building these theories, this study aims to develop a prognostic model based on differential autophagy genes associated with HNSC and validate its value.

## Methods

### Data collection

Gene Expression Omnibus (GEO, https://www.ncbi.nlm.nih.gov/geo/)^3^ is a public genomics database with a large number of high-throughput gene expression data and related information. The Cancer Genome Atlas (TCGA, https://www.cancer.gov/about-nci/organization/ccg/research/structural-genomics/tcga) is the largest database of cancer genetic information currently, with rich and standardized clinical data and a large number of samples for each type of cancer. Human Autophagy Database (HADb, autophagy.lu/index.html) is a publicly available database for autophagy genes, housing a wealth of relevant information regarding human autophagy genes. We downloaded the description information of series matrix files and data sheet about the expression profile dataset GSE112026,^4^ GSE41613^5^ and GSE65858^6^ of the patients with head and neck squamous cell carcinoma from GEO and filter the data of HNSC samples and control samples. GSE112026 is Homo sapiens which sequencing platform is GPL16791 Illumina HiSeq 2500 (Homo sapiens). We selected 47 HNSC samples and 25 control samples for this study. GSE41613 is Homo sapiens which sequencing platform is GPL570 [HG-U133_Plus_2] Affymetrix Human Genome U133 Plus 2.0 Array including 97 HNSC samples and its clinical messages. GSE65858 is Homo sapiens which sequencing platform is GPL10558 Illumina HumanHT-12 V4.0 expression beadchip including 270 HNSC samples and its clinical messages. We downloaded TPM gene expression matrix data, clinical information, somatic mutation data and copy number variation data of TCGA-HNSC from TCGA. TCGA-HNSC has 312 HNS samples meeting the analysis requirements of this study. 679 HNSC samples from GSE41613 GSE65858 and TCGA-HNSC contained the clinical features of survival status, survival time, patient age, patient sex and HNSC pathological stage for subsequent subtype analysis and prognosis analysis. The autophagy-related genes required for our study were downloaded from HADb.

### Data preprocessing

Data preprocessing involves the transformation from probes to gene symbols, data integration, and batch normalization. Probes without gene symbols were removed, and the average expression level was calculated for genes with multiple probes. Batch effects were eliminated from the merged data using the ComBat function from the *R* package sva.

### Selection of differentially expressed genes associated with HNSC disease

The gene expression matrix of dataset GSE112026 was used as the subject of analysis. The R package limma (version 3.50.0)^7^ was employed to perform differential gene expression analysis and identify genes with dysregulated expression in HNSC disease. The criteria for filtering Differentially Expressed Genes (DEGs) were set as |logFC| > 0.5 and adj.P.Val < 0.05. Genes with logFC > 0.5 and adj.P.Val < 0.05 were considered as up-regulated DEGs, while genes with logFC < −0.5 and adj.P.Val < 0.05 were considered as down-regulated DEGs.

To obtain the Autophagy-related differentially expressed genes in HNSC disease, we took the intersection of DEGs and autophagy-related genes, where the overlapping genes represented the Autophagy-related DEGs.

### Functional enrichment analysis of autophagy related DEGs

We performed Gene Ontology (GO) and Genes and Genomes (KEGG) enrichment analysis on the Autophagy-related DEGs using the *R* package clusterProfiler.^8^ The criteria for selecting significant entries were set as p < 0.05 and a False Discovery Rate (FDR) value (*q*-value) < 0.05, which were considered statistically significant. The Benjamini-Hochberg (BH) method was applied for p-value correction.

### Analysis of tumor module-associated differential autophagy genes

For the module most correlated with HNSC disease in the Weighted Correlation Network Analysis (WGCNA)^9^ analysis, the genes within this module were intersected with the Autophagy-related DEGs to obtain the tumor module-associated differential autophagy genes. Subsequently, using the somatic mutation data of HNSC obtained from TCGA, we generated a waterfall plot illustrating the mutations in the tumor module-associated differential autophagy genes. Additionally, based on the copy number variation data of TCGA-HNSC, we compiled amplification and deletion information and depicted a lollipop plot showing the amplifications and deletions of the tumor module-associated differential autophagy genes.

### Constructing molecular subtypes based on correlated differential autophagy genes in tumor modules

Consensus Clustering is an algorithm based on resampling used to identify each member and its subgroup number and verify the rationality of the clustering. The multiple iterations of consistent clustering on the subsamples of the dataset provide indicators of clustering stability and parameter decision by inducing sampling varia-bility through subsampling. We used 679 HNSC disease samples from three datasets (GSE41613, GSE65858 and TCGA-HNSC) which contained survival status, survival time, patient age, patient gender and clinical characteristics of HNSC pathological stage, and took the combined gene expression matrix of these samples as the analysis object. The consensus clustering method of Consensus Cluster Plus^10^ from *R* package was used to identify different HNSC disease subtypes based on tumor module-related differential autophagy genes. We used Kaplan-Meier survival curve analysis to analyze the HNSC disease subtype OS (Overall Survival), and the compared survival difference. In addition, we analyzed gene expression differences among HNSC disease subtypes.

### Gene set enrichment analysis

GSEA (Gene Set Enrichment Analysis)^11^ used to evaluate a set of predefined genetic gene in the table and the sorting of phenotypic correlation gene distribution trend, so as to determine its contribution to the phenotype. In this study, *R*-package cluster Profiler was used for enrichment analysis of all genes sequenced according to logFC. The parameters used in the GSEA enrichment analysis were as follows: the number of calculations was 1000, each gene set contained a minimum of 10 genes and a maximum of 500 genes, and the p-value correction method was Benjamini-Hochberg (BH). C2.cp.kegg.v7.2.symbols gene set was obtained from Molecular Signatures Database (MSigDB),^12^ and the screening criterion for significant enrichment was p-value < 0.05.

### Construction of prognosis model

Further determination of the prognostic value of tumor module-associated differential autophagy genes was conducted by performing survival analysis and integrating gene expression data and clinical information from 679 HNSC samples. Initially, a survival analysis was performed on the tumor module-associated differential autophagy genes to identify genes significantly associated with survival (p-value < 0.05). Subsequently, Boruta feature selection was employed to confirm the key prognostic genes. The dataset of the 679 HNSC samples was then randomly divided into a training set (70% of the samples) and a validation set (30% of the samples) to construct the prognostic model. The important genes identified by the Boruta feature selection were used in a multivariable Cox regression analysis to build the prognostic model. The risk score calculation formula is as follows:

$$\text{r}\text{i}\text{s}\text{k}\text{S}\text{c}\text{o}\text{r}\text{e}=\sum_{\text{i}=1}^{\text{n}}\text{C}\text{o}\text{e}\text{f}({\text{g}\text{e}\text{n}\text{e}}_{\text{i}})\text{*}\text{E}\text{x}\text{p}\text{r}\text{e}\text{s}\text{s}\text{i}\text{o}\text{n}({\text{g}\text{e}\text{n}\text{e}}_{\text{i}})$$

Coef (gene_i_) represents multi-factor Cox regression coefficient, expression (gene_i_) represents the expression value of each gene, and n represents the number of genes.

The 679 HNSC disease samples were divided into high-risk and low-risk groups based on the median risk score of the training set. Kaplan-Meier survival curve was used for OS analysis and time-dependent ROC analysis to evaluate the prediction accuracy of the model.

### Analysis of prognostic genes

In this study, GeneMania database was used to construct protein interaction networks of prognostic genes.

For the obtained prognostic genes, we queried the microRNA that interact with them from miRDB database, miRTarbase database and starBase database and take the intersection of the query results of these three databases as the microRNA that interacts with the prognostic genes. We queried lncRNA that interact with the above microRNA in the starBase database. Finally, based on the above query results, we used Cytoscape to draw the ceRNA network diagram.

### Experimental cells and main reagents

The human laryngeal cancer cell line TU686 was purchased from Hunan Fenghui Biotechnology Co., Ltd., and the human tongue squamous cell Carcinoma Cell Line (CAL-27) was purchased from Sevier Biotechnology; The SYBR Green Pro Taq HS pre mixed qPCR kit was purchased from Aikerui Biotechnology; Fetal bovine serum, trypsin (0.25% Trypsin EDTA, containing phenol red), DMDM medium, and 1640 medium were purchased from Gibco Corporation in the United States; Phosphate Buffered Saline Solution (PBS) was purchased from Fetal Bovine Serum (FBS) Biosharp company; Evo M-MLV reverse transcription premix kit (including gDNA removal reagent for qPCR) Ver.2 was purchased from Hunan Aikeli Bioengineering Co., Ltd; Lipofectamine 2000 transfection reagent was purchased from Thermo Fisher Scientific.

### Real-time RT-PCR

We used an RNA extraction kit to extract total RNA from cells, reverse transcribed and synthesized cDNA, and performed RT qPCR using a real-time fluorescence quantitative PCR instrument (ABI, 7500 Fast, USA). We used β-actin (forward sequence 5'-TGGCACCCAGCACAATGAA-3'), reverse sequence 5'-CTAAGTCATAGTCCTACGCCTAGAGAGAA-3'), human GAPDH (forward sequence 5'-GCACCGTCAAGTGGAAC-3'), and reverse sequence 5'-TGGTGAGAACGCCAGTGGGAA-3') as references to amplify genes targeting GSK3B (forward sequence 5'-GTTTTCGGTACTATAGCACCA-3', reverse sequence 5'- TCTACAGCTCAGCCAACA-3').

### Wound healing assay

We seeded the cells into a 6-well plate and transfected them after the cell density reached 100% and performed scratch test operation after transfection is completed. We also rinsed the scratch area with 1 × PBS, then we observed and record the scratch healing under a microscope at 0-, 24-, and 48-h.

### Cell transfection experiment

General Bio (Anhui) Co., Ltd is responsible for designing and synthesizing SiRNA-GSK3B. The experimental group includes SiRNA-GSK3B1, SiRNA-GSK3B2, SiRNA-GSK3B3 groups, and the negative control group is the NC group. We inoculate the cells into a 6-well plate, and when the cell density reaches 60%–80%, slowly drip the pre prepared transfection system into the 6-well plate and incubate in a 37, 5% CO_2_ incubator for 48-h. RT-qPCR detects knockdown efficiency.

### Immunohistochemical experiment

Firstly, we fixed the tissue samples in 10% formalin for approximately 24 h; next, we sliced the case into 5 μm sections and deparaffinized twice with xylene for 10 min each time, gradually hydrating to PBS; then, we used citric acid buffer and incubated at high temperature of 95 °C for 30 min to recover the antigen, and blocked the non-specific binding site with 5% normal serum for 30 min; we added 1:200 primary antibody to the slices and incubate overnight at 4 °C; after washing away the primary antibody, we added a secondary antibody that matches the primary antibody and incubate at room temperature for 1 h; we performed DAB color reaction for about 5–10 minutes until a brown precipitate appears; Finally, we used H&E staining for comparison, and the staining results were observed and analyzed it.

### Statistical method

We used R4.1.2 and GraphPad Prism 9 statistical software to analyze the data. Quantitative data is expressed as (x ± s) and subjected to analysis of variance and *t*-test; Perform a Chi-Square test for comparison of count data; p < 0.05 is considered statistically significant.

## Results

### Autophagy-related DEGs in HNSC samples

**Fig. 1:**
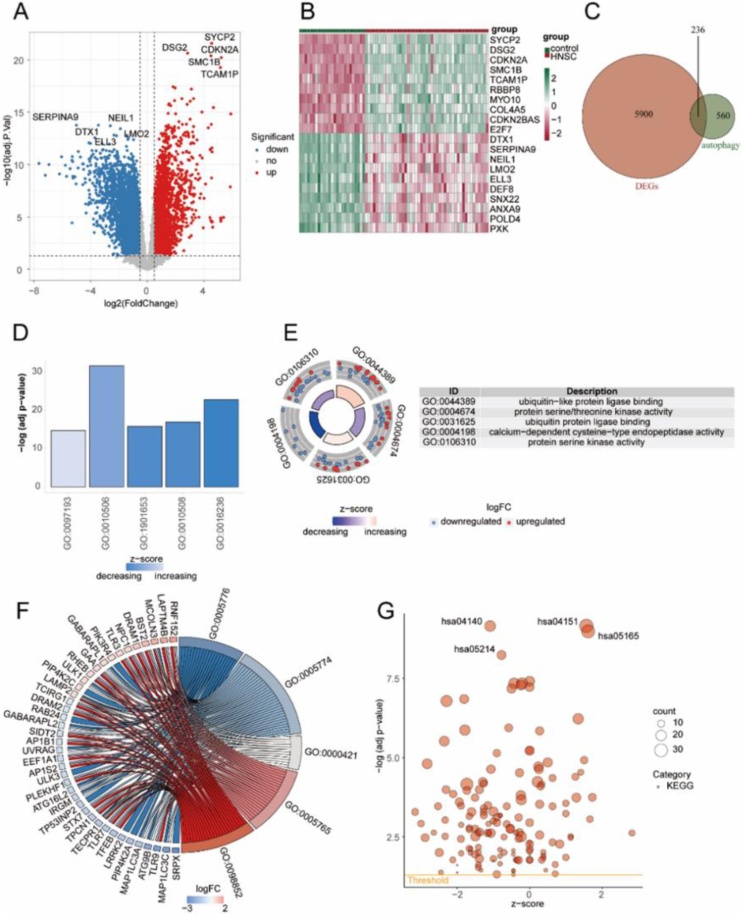
Autophagy-related genes with differential expression in HNSC disease. (A) Volcano plot showing the differentially expressed genes between HNSC disease samples and control samples. (B) Heatmap depicting the expression distribution of the top 20 DEGs between HNSC disease samples and control samples. (C) Venn diagram illustrating the overlap between DEGs and Autophagy-related genes. (D) Bar plot displaying the Biological Process (BP) pathways in the results of the GO enrichment analysis. (E) Circular plot representing the Molecular Function (MF) pathways in the results of the GO enrichment analysis; the outer scatter plot represents individual genes, and the inner bar plot represents the significance of the corresponding enrichment results. (F) Chord plot illustrating the Cellular Component (CC) pathways in the results of the G0 enrichment analysis, with genes on the left and the enriched pathways on the right. (G) Bubble plot representing the results of the KEGG enrichment analysis.

In this project, gene expression difference analysis was conducted between HNSC samples and control samples, and 6163 differentially expressed genes was obtained, among which 2504 genes were up-regulated (logFC > 0.05, adj.P.V < 0.05).The expression of 3632 basal factors was down-regulated (logFC < −0.5, adj.P.V < 0.05). Volcano plot was used to show the results of gene expression difference analysis, and 5 most significant genes were selected from each of the down-regulated genes for labeling. Heat maps were drawn to visually analyze the expression distribution of top20 differentially expressed genes in HNSC samples and control samples (Fig. 1A). The expression levels of genes SYCP2, DSG2, CDKN2A, SMC1B, TCAM1P, RBBP8, MYO10, COL4A5, CDKN2BAS and E2 F7 in HNSC samples were lower than those in control group. The expression levels of genes DTX1, SERPINA9, NEIL1, LMO2, ELL3, DEF8, SNX22, ANXA9, POLD4 and PXK in HNSC samples were higher than those in control group (Fig. 1B). The intersection between DEGs and Autophagy-related genes was analyzed using Venn diagram, and 236 identical genes were found between them. The intersection genes were regarded as Autophagy-related DEGs (Fig. 1C).

We performed GO functional enrichment analysis and KEGG functional enrichment analysis for Autophagy-related DEGs present in HNSC samples. For the GO functional enrichment analysis, combined with the logFC value to calculate the corresponding z-score value of each gene, and the enriched Biological Process (BP) pathway was visualized with the histogram. It was found that the enriched BP pathways were mainly composed of intrinsic apoptotic signaling pathways (GO: 0097193), regulation of autophagy (GO: 0010506), cellular response to peptide (GO: 1901653), positive regulation of autophagy (GO: 0010508), macroautophagy (GO: 0016236) and so on (Fig. 1D). The enriched Molecular Function (MF) pathway was visually analyzed with a loop diagram. It was found that the enriched MF pathway was mainly composed of ubiquitin-like protein ligase binding (GO: 0044389), protein serine/threonine kinase activity (GO: 0004674), ubiquitin protein ligase binding (GO: 0031625), calcium-dependent cysteine-type endopeptidase activity (GO: 0004198), protein serine kinase activity (GO: 0106310) and so on (Fig. 1E). The enriched Cellular Component (CC) pathways were visually analyzed with string diagram. It was found that the enriched CC pathways mainly included autophagosome (GO: 0005776), vacuolar membrane (GO: 0005774), vacuolar membrane (GO: 0005774), autophagosome membrane (GO:0000421), lysosomal membrane (GO:0005765), lytic vacuole membrane (GO: 0098852) and so on (Fig. 1F). KEGG functional enrichment analysis found that Autophagy-related DEGs in HNSC disease samples were mainly enriched in Autophagy-animal (hsa04140), PI3K-Akt signaling pathway (hsa04151), Human papillomavirus infection (hsa05165), Glioma (hsa05214) and so on (Fig. 1G).

### WGCNA analysis

**Fig. 2:**
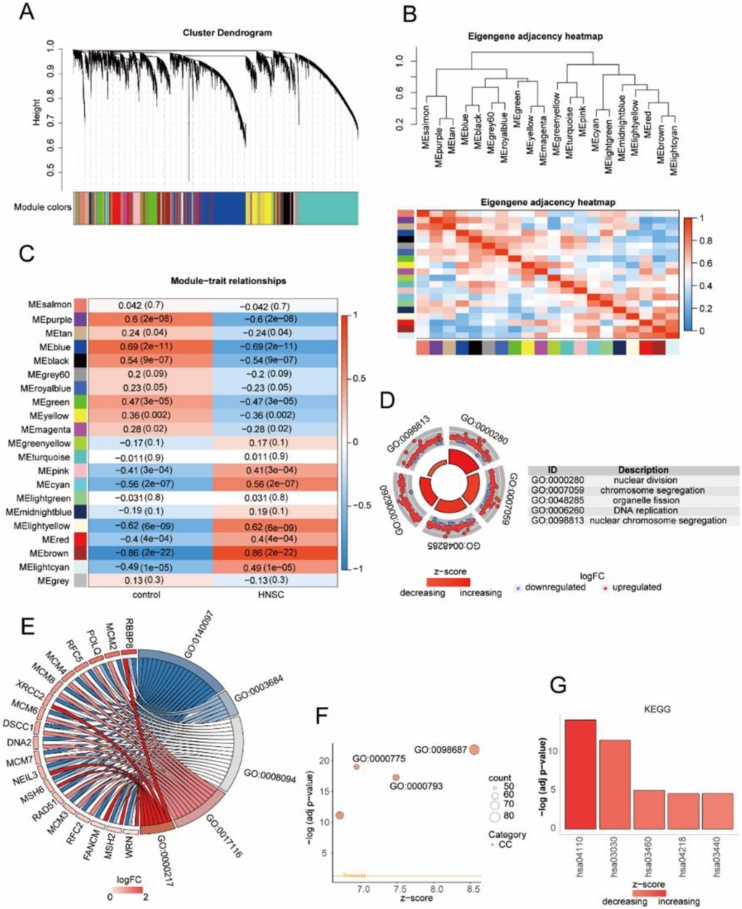
Identification of gene modules most associated with HNSC disease. (A) Dendrogram of gene clustering. (B) Heatmap of module assignment. (C) Heatmap depicting the correlation between HNSC disease samples, control samples, and the WGCNA gene clustering modules. (D) Circular plot showing the results of the GO enrichment analysis of the modules associated with tumor-related genes; the outer scatter plot represents individual genes, and the inner bar plot represents the significance of the corresponding enrichment results for Biological Process (BP) pathways. (E) Chord plot illustrating the Molecular Function (MF) pathways in the results of the GO enrichment analysis for the module associated with tumor-related genes, with genes on the left and the enriched pathways on the right. (F) Bubble plot representing the results of the Cellular Component (CC) pathways in the GO enrichment analysis for the modules associated with tumor-related genes. (G) Bar plot displaving the results of the KEGG enrichment analysis for the modules associated with tumor-related genes.

In this study, WGCNA was used to construct a co-expression network and search for gene modules highly associated with HNSC disease. We performed WGCNA analysis on the control samples and HNSC disease samples, and constructed a hierarchical clustering tree according to the correlation coefficients among genes, dividing the genes into 21 modules (Fig. 2A). We also used heat maps to visualize correlations among gene modules (Fig. 2B). The correlation between HNSC disease samples and control samples and the above 21 modules was calculated and visualized. The results showed that brown module had the highest correlation with HNSC disease (Fig. 2C). We performed GO functional enrichment analysis and KEGG functional rich set analysis for clustered genes in the brown module. For the GO functional enrichment analysis, the *z*-score value corresponding to each gene was calculated in combination with the logFC value, and the enriched Biological Process (BP) pathway was visually analyzed with a loop diagram. It was found that the enriched BP pathways mainly include nuclear division (GO: 0000280), chromosome segregation (GO: 0007059), organelle fission (GO: 0048285) and DNA replication (GO: 0006260), nuclear chromosome segregation (GO: 0098813) and so on. (Fig. 2D). The enriched Molecular Function (MF) pathway was visually analyzed by string diagram. It was found that the enriched MF pathway mainly consisted of catalytic activity, acting on DNA (GO: 0140097), damaged DNA binding (GO: 0003684), ATP-dependent activity, acting on DNA (GO: 0008094), single-stranded DNA helicase activity (GO: 0017116), DNA secondary structure binding (GO: 0000217) and so on. (Fig. 2E). The enriched Cellular Component (CC) pathway was visually analyzed by Bubble diagram. It was found that the enriched CC pathways mainly included chromosomal region (GO:0098687), chromosome, centromeric region (GO:0000775), condensed chromosome (GO: 0000793) and so on (Fig. 2F). The KEGG functional enrichment analysis results visually was analyzed by histogram. It was found that the enriched pathways mainly included Cell cycle (hsa04110), DNA replication (hsa03030), Fanconi anemia pathway (hsa03460) and Cellular senescence (hsa04218), Homologous recombination (hsa03440) and so on (Fig. 2G).

### The analysis of tumor-module-associated differential autophagy genes

**Fig. 3:**
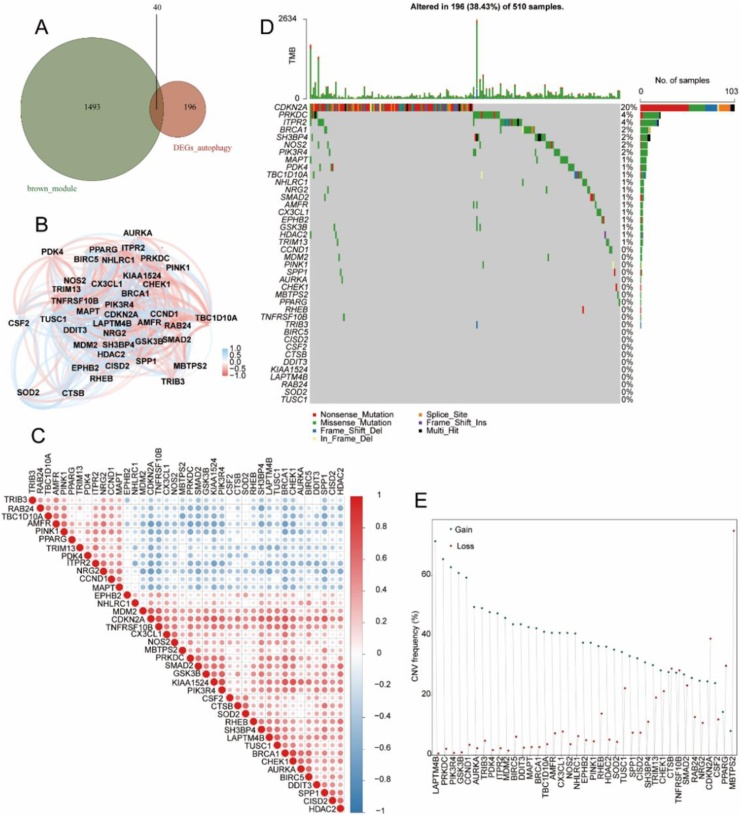
Analysis of tumor module-associated diferential autophagy genes. (A) Venn diagram illustrating the overlap between genes in the brown module and Autophagy-related Differential Expression Genes (Autophagy-related DEGs) in HNSC disease. (B) Network plot showing the correlation among tumor module-associated diferential autophagy genes. (C) Heatmap displaying the correlation among tumor module-associated differential autophagy genes. (D) Oncoplot depicting the somatic mutation landscape of the tumor module-associated differential autophagy genes in TCGA-HNSC. The upper bar plot shows the overall mutation frequency per sample, and the stacked bar plot on the right side represents the proportion of each variant type. (E) CNV freauency plot of the tumor module-associated differential autophagy genes in TCGA-HNSC.

According to the results of WGCNA analysis, brown module has the highest correlation with HNSC disease. We analyzed the intersection between clustered genes in brown module and Autophagy-related DEGs in HNSC disease with Wayne diagram, and found that the two have 40 identical genes, namely AMFR, AURKA, BIRC5, BRCA1, CCND1, CDKN2A, CHEK1, CISD2, CSF2, CTSB, CX3CL1, DDIT3, EPHB2, GSK3B, HDAC2, ITPR2, KIAA1524, LAPTM4B, MAPT, MBTPS2, MDM2, NHLRC1, NOS2, NRG2, PDK4, PIK3R4, PINK1, PPARG, PRKDC, RAB24, RHEB, SH3BP4, SMAD2, SOD2, SPP1, TBC1D10A, TNFRSF10B, TRIB3, TRIM13 and TUSC1. These intersection genes were identified as tumor-module-associated differential autophagy genes in HNSC diseases (Fig. 3A). We also analyzed the correlation of tumor-module-related differential autophagy genes’ expressions. It was found that genes TRIB3, RAB24, TBC1D10A, AMFR, PINK1, PPARG, TRIM13, PDK4, ITPR2, NRG2, CCND1 and MAPT were negatively correlated with multiple gene expressions (Fig. 3B‒C). For the above tumor mode-related differential autophagy genes, 196 of 510 TCGA-HNSC samples had gene mutations, and the mutation frequency were 38.43%. The results showed that gene CDKN had the highest mutation frequency (20%), followed by genes PRKDC and ITPR2, and the mutation frequency were 4% (Fig. 3D). For the above tumor-modular-related differential autophagy genes, 39 of them were present in CNV data of TCGA-HNSC, and the exploration of copy values revealed abnormal changes in these genes. The genes CTSB, TNFRSF10B, CDKN2A, PPARG and MBTPS2 showed CNV loss (Fig. 3E).

### The analysis of the molecular subtypes of tumor-module-related differential autophagy genes in HNSC

**Fig. 4:**
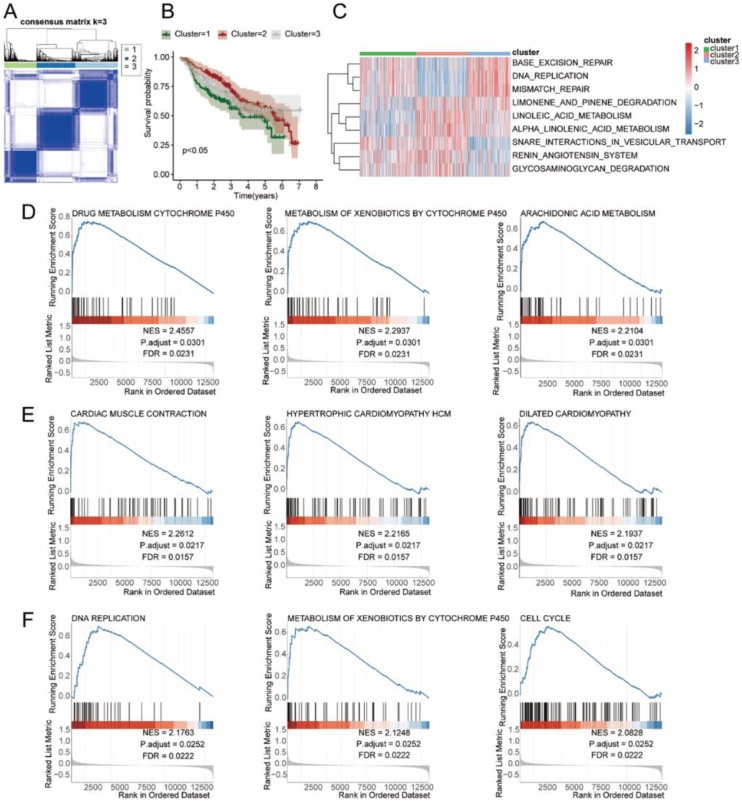
Identification of three molecular subtypes related to tumor module-associated differential autophagy genes in HNSC using Consensus Clustering. Subtype analysis. (A) Heatmap of Consensus Clustering with *k* = 3, where the values in the Consensus matrix range from 0 (unlikely to clustertogether) to 1 (always cluster together) and are represented by white to dark blue. (B) Survival curvesof the three subtypes in HNSC, where p < 0.05 indicates significant differences in overall survivalbetween subtypes. (C) Heatmap displaying the distribution of enrichment scores for significantlyenriched pathways based on GSVA analysis in HNSC samples grouped by subtypes. (D) Significantly differentially enriched pathways between Cluster 1 as a separate group and Clusters 2 and 3 asanother group. (E) Significantly differentially enriched pathways between Cluster 2 as a separate group and Clusters 1 and 3 as another group. (F) Significantly differentially enriched pathways between Cluster 3 as a separate group and Clusters 1 and 2 as another group.

We distinguished HNSC disease samples subtypes based on tumor-mode-related differential autophagy genes. Through consensus cluster analysis, the optimal cluster number was determined to be 3, and the HNSC disease samples were divided into three subtypes: Cluster 1, Cluster 2 and Cluster 3 (Fig. 4A). Survival analysis of the three subtypes showed significant differences in the overall survival of the three HNSC disease samples (p-value < 0.05, Fig. 4B). Heat maps were used to show the distribution of enrichment fraction of gene enrichment pathways in subtypes of HNSC disease samples which obtained through GSVA analysis (Fig. 4C). GSEA enrichment analysis was performed on HNSC disease sample subtypes, Cluster 1 as a group, Cluster 2 and Cluster 3 as a group. The pathway with significant gene enrichment difference between them is DRUG_METABOLISM_CYTOCHROME_P450 (NES = 2.4557, P.adjust = 0.0301, FDR = 0.0231), METABOLISM_OF_XENOBIOTICS_BY_CYTOCHROME_P450 (NES = 2.2937, P.adjust = 0.0301, FDR = 0.0231), ARACHIDONIC_ACID_METABOLISM (NES = 2.2104, P.adjust = 0.0301, FDR = 0.0231) and so on (Fig. 4D). Cluster 2 as a separate group, Cluster 1 and Cluster 3 as a separate group, and the pathways with significant gene enrichment differences between them were CARDIAC_MUSCLE_CONTRACTION (NES = 2.2612, P.adjust = 0.0217, FDR = 0.0157), HYPERTROPHIC_CARDIOMYOPATHY_HCM (NES = 2.2165, P.adjust = 0.0217, FDR = 0.0157), DILATED_CARDIOMYOPATHY (NES = 2.1937, P.adjust = 0.0217, FDR = 0.0157) and so on (Fig. 4E). Cluster 3 as a separate group, Cluster 1 and Cluster 2 as a separate group, and the pathways with significant gene enrichment differences between them were DNA_REPLICATION (NES = 2.1763, P.adjust = 0.0252, FDR = 0.0222), METABOLISM_OF_XENOBIOTICS_BY_CYTOCHROME_P450 (NES = 2.1248, P.adjust = 0.0252, FDR = 0.0222) CELL_CYCLE (NES = 2.0828, P.adjust = 0.0252, FDR = 0.0222) and so on (Fig. 4F).

### Subtype difference analysis of tumor-module-related differential autophagy genes

**Fig. 5:**
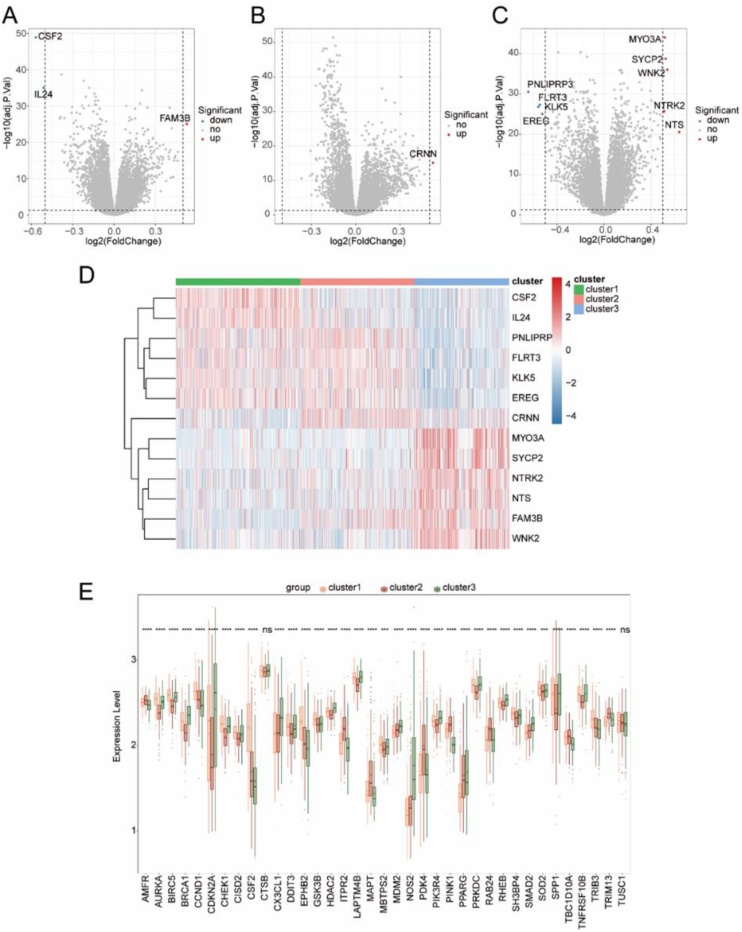
Analysis of subtype differences in tumor module-associated differentia autophagy genes. (A) Volcano plot depicting the differentially expressed genes between Cluster 1 as a separate group and Clusters 2 and 3 as another group in HNsC disease samples. (B) Volcano plot depicting the differentially expressed genes between Cluster 2 as a separate group and Clusters 1 and 3 as another group inHNSC disease samples. (C) Volcano plot depicting the differentially expressed genes between Cluster 3 as a separate group and Clusters 1 and 2 as another group in HNSC disease samples. (D) Heat map displaying the expression distribution of genes differentially expressed between HNsC disease sub-groups in each sample. (E) Boxplot illustrating the differential expression of tumor.

According to the consistent clustering results, HNSC disease samples were classified into Cluster 1, Cluster 2 and Cluster 3 categories. Cluster 1 as a single group, Cluster 2 and Cluster 3 as a single group, three differentially expressed genes were identified between them, and one gene was up-regulated (logFC > 0.5, adj. p.AL < 0.05). The two genes were down-regulated (logFC < -0.5, adj.P.Val < 0.05). The results of gene expression difference analysis were shown by volcano map, and the genes with significant differences were labeled (Fig. 5A). Cluster 2 as a single group, Cluster 1 and Cluster 3 as a single group, they identified one differentially expressed gene, and the expression of this gene was up-regulated (logFC > 0.5, adj.P.Val < 0.05). The results of gene expression difference analysis were shown by volcano plot. The genes with significant differences were labeled (Fig. 5B). Cluster 3 as a single group, Cluster 1 and Cluster 2 as a single group, they identified 9 differentially expressed gene, 5 genes were up-regulated (logFC > 0.5, adj. p.AL < 0.05), and 4 genes were down-regulated (logFC < -0.5, adj.P.Val < 0.05). The results of gene expression difference analysis were shown by volcano plot, and the genes with significant differences were labeled (Fig. 5C). We used heat maps to visualize the expression distribution of the above identified differentially expressed genes in HNSC disease samples (Fig. 5D). Box plot was used to demonstrate the differential expression of tumor-module-related differential autophagy genes among subtypes. We found genes AMFR, AURKA, BIRC5, BRCA1, CCND1, CDKN2A, CHEK1, CISD2, CSF2, CX3CL1, DDIT3, EPHB2, GSK3B, HDAC2, ITPR2, KIAA1524, LAPTM4B, MAPT, MBTPS2, MDM2, NHLRC1, NOS2, NRG2, PDK4, PIK3R4, PINK1, PPARG, PRKDC, RAB24, RHEB, SH3BP4, SMAD2, SOD2, SPP1, the expressions of TBC1D10A, TNFRSF10B, TRIB3 and TRIM13 were significantly different among the three subtypes of HNSC disease samples, while the expressions of CTSB and TUSC1 were not significantly different among the three subtypes of HNSC disease samples (Fig. 5E).

### Construction of HNSC prognostic model

**Fig. 6:**
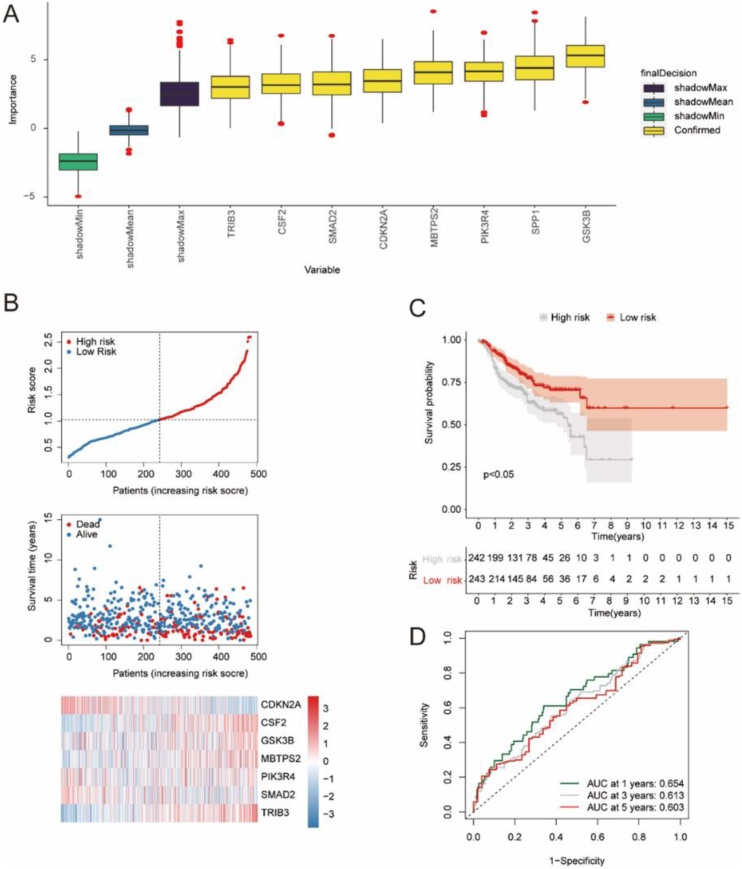
The prognosis model of HNSC disease was constructed based on tumor-module-associated differential autophagy genes. (A) Boruta characteristics screened out 8 important prognostic genes. The blue box plot corresponds to the minimum, average, and maximum Z-scores of a shadedproperty, and the yellow box plot represents the Z-scores of the confirmed property. (B) The trainingset of risk triptych. The upper picture: The risk curve of the training set. The medium one: Thescatter-plot of survival state of the training set. The lower one: Prognostic gene expression heatmap in training set, behavioral genes, list samples in order of risk score from smallest to largest. (C) Kaplan-Meier survival analysis for the high-low-risk group of the training set. p < 0.05 indicates that there is a significant difference in OS between high and low risk groups. (D) 1-year, 3-year and 5-year ROC curves calculated from risk scores for the training set.

Survival analysis was performed on the tumor module-associated differ-rential autophagy genes in the HNSC disease sample dataset, leading to the preliminary identification of 23 genes associated with prognosis: AURKA, CDKN2A, CHEK1, CSF2, CX3CL1, DDIT3, EPHB2, GSK3B, HDAC2, ITPR2, MBTPS2, MDM2, NOS2, PDK4, PIK3R4, PINK1, PRKDC, RAB24, SMAD2, SOD2, SPP1, TRIB3 and TUSC1 (p-value < 0.05). Then, eight important prognostic genes were identified by using Boruta feature screening: CDKN2A, CSF2, GSK3B, MBTPS2, PIK3R4, SMAD2, SPP1 and TRIB3 (Fig. 6A). Finally, we constructed a prognostic model by using multivariate Cox regressive analysist, and calculated Cox regression coefficients of prognostic genes. According to the collinearity detection results, 7 genes were included in the prognostic model, namely CDKN2A, CSF2, GSK3B, MBTPS2, PIK3R4, SMAD2 and TRIB3. We also passed the risk score = (−0.38) * CDKN2A + 0.267 * CSF2 + 0.524 * GSK3B + 0.916 * MBTPS2 + 0.072 * PIK3R4 + (-0.318) * SMAD2 + 1.282* TRIB3 to calculate risk scores and divided HNSC disease samples into low risk and high risk groups based on the median value of the risk scores in the training set (Training set: 242 high risk cases and 243 low risk cases; Validation set: 105 high risk cases and 89 low risk cases). We presented the risk triptych for the training set (Fig. 6B). Survival analysis results showed that the training set had significant differences in OS between high and low risk groups (p-value < 0.05) (Fig. 6C). The 1-year, 3-year and 5-year AUC of the risk scores based on the prognostic model in the training set were all greater than 0.6, indicating that the prognostic model had certain prognostic value (Fig. 6D).

### Validation of HNSC disease prognostic model and protein interaction network and cerna network of prognostic genes

**Fig. 7:**
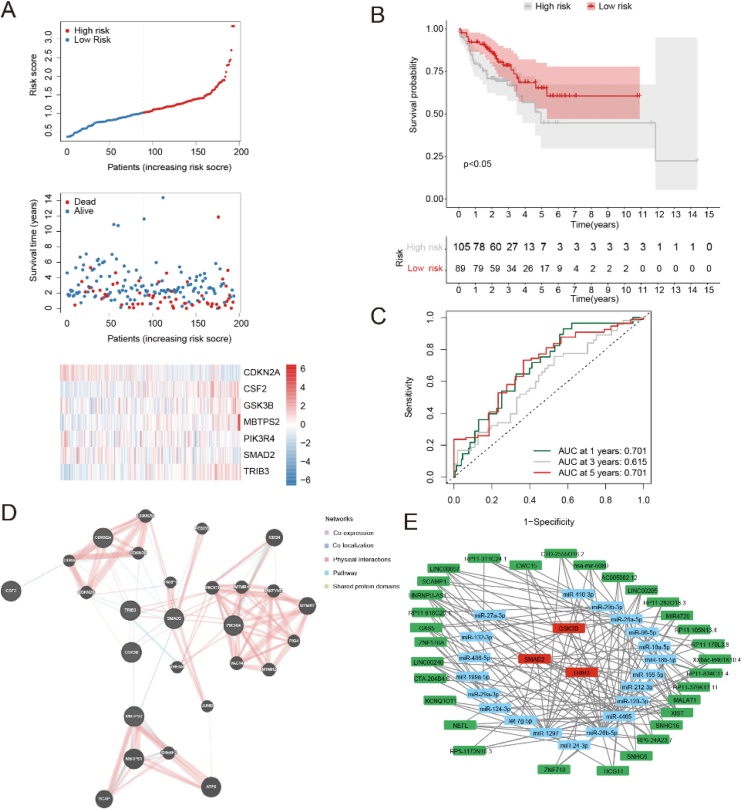
Validation of HNSC disease prognosis model and protein interaction network and ceRNA network of prognostic genes. (A) Risk triplets of validation sets. The upper picture. The risk curve of the validation set. The medium one: the scatterplot of survival state of the validation set. The lower one: Prognostic gene expression heat map in validation set, behavioral genes, istsamples in order of risk score from smallest to largest. (B) Kaplan-Meier survival analysis for the high-low-risk group of the validation set, p < 0.05 indicates that there is a significant difference in OS between high and low risk groups. Gray represents the high-risk group and red represents the low-risk group. (C) 1-year, 3-year and 5-year ROC curves calculated from risk scores for the validation set. (D) PPl networks. (E) ceRNA network diagram among prognostic genes, microRNA, and IncRNA. Red represents prognostic genes, blue microRNA, and green IncRNA.

We show the risk triptych for the validation set (Fig. 7A). Survival analysis results showed that there was a significant difference in OS in the validation set between the high and low risk groups (p-value < 0.05) (Fig. 7B). The 1-year, 3-year and 5-year AUC of the risk scores based on the prognostic model in the validation set were all greater than 0.6, which further verified that the prognostic model had certain prognostic value (Fig. 7C). We analyzed the protein interaction network of prognostic genes (CDKN2A, CSF2, GSK3B, MBTPS2, PIK3R4, SMAD2 and TRIB3) and used the tool GeneMania to construct a protein interaction network composed of 27 genes. The genes in this protein interaction network are CSF2, CDK6, CDKN2A, CDKN2B, CDKN2D, CDKN2C, FOXO3, PARP1, TRIB3, SMAD2, GSK3B, THEM4, JUNB, RBSN, PIK3C3, MTMR4, PIKFYVE, MTMR7, PIK3R4, FIG4, VAC14, MTMR2, MBTPS2, MBTPS1, SREBF2, SCAP, ATF6 (Fig. 7D). For the above 7 prognostic genes, we screened 20microRNA targeting and 3 prognostic genes of them. Then, we predicted 20 screened microRNA targeting lncRNA, and 31 lncRNA were screened. The ceRNA network map was drawn to visually analyze the network relationships between prognostic genes, microRNA and lncRNA (Fig. 7E).

The effect of knocking down GSK3B expression on the migration ability of TU686 and CAL-27 cells

**Fig. 8:**
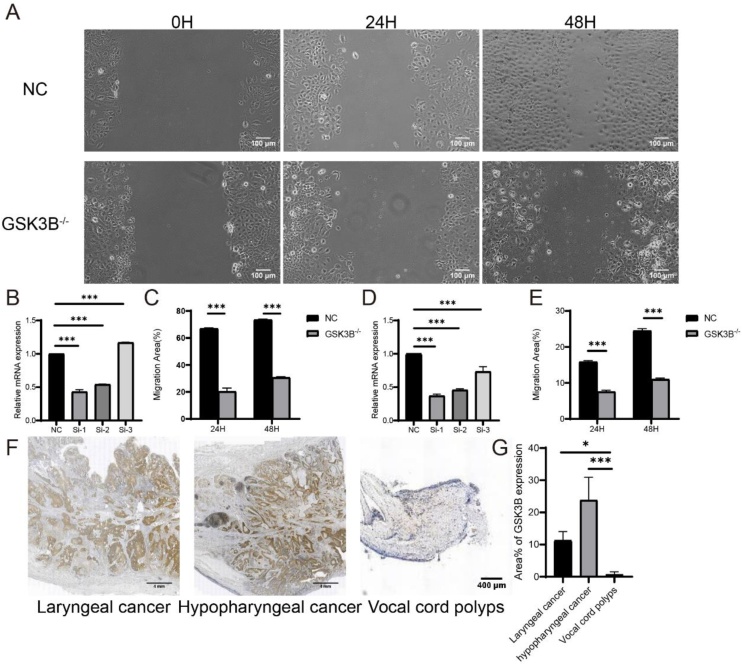
(A) Untreated and GSK3B knockdown TU686 were subjected to cell transfection experiment, and the cells were photographed under bright conditions at 0 h, 24 h, and 48 h. (B) The comparison of GSK3B-related expression levels between TU686 cells transfected with SiRNA-GSK3B1, SiRNA-GSK3B2, and SiRNA-GSK3B3, and the NC group cells. (C) The migratory ability of TU686 cells was observed at 24- and 48-h, comparing untreated groups and those transfected with SiRNA-GSK3B. (D) The migratory ability of CAL-27 cells was observed at 24- and 48-h, comparing untreated groups and those transfected with SiRNA-GSK3B. (E) The migratory ability of CAL-27 cells was observed at 24 and 48 h, comparing untreated groups and those transfected with SiRNA-GSK3B. (F) The images of laryngeal cancer, pharyngeal cancer, and vocal cord polyps after IHC. (G) The proportion of GSK3B expression area in three types of tissues.

We transfected SiRNA-GSK3B1, SiRNA-GSK3B2, and SiRNA-GSK3B3 into TU686 and CAL-27 cells respectively, then we extracted and synthesized cDNA. After RT-qPCR experiments, we found that the expression level of GSK3B was significantly decreased in the SiRNA-GSK3B1 and SiRNA-GSK3B2 groups (ALL p < 0.05, Fig. 8B showed the expression level of SiRNA-GSK3B in TU686; Fig. 8D showed the expression level of Si-RNAGSK3B in CAL-27), but the transfection efficiency of GSK3B1 was the highest. So, we chose SiRNA-GSK3B1 for post-experiments. The wound healing assay results showed that compared with the NC group, knocking down GSK3B significantly reduced the migration ability of TU686 and CAL-27 cells (all p < 0.05, Fig. 8A, C show the TU686 result, and Fig. 8E shows the CAL-27 result). We took pathological sections from 30 patients with laryngeal cancer, 30 patients with pharyngeal cancer, and 10 patients with vocal cord polyps, and performed immunohistochemical DAB staining on them. After staining, we found that the proportion of GSK3B in laryngeal cancer and pharyngeal cancer was significantly higher than that in vocal cord polyps, indicating that the expression of GSK3B is high in cancer tissues and indirectly plays an important role in tumor growth (Fig. 8F‒G).

## Discussion

HNSC being the 6th most common cancer globally, have attracted considerable attention in terms of diagnosis and treatment. Autophagy plays a dual role in tumor development. Therefore, in this study, a prognostic model was constructed based on differential autophagy genes associated with the tumor module in HNSC. The value of this prognostic model was tested using HNSC disease samples. Based on the calculated risk scores, the HNSC samples were divided into high- and low-risk groups, and survival analyses were performed on the training and validation sets. The results showed significant differences in OS between the high- and low-risk groups in both the training and validation sets, indicating that the risk-based grouping in this study had a certain basis and could effectively categorize the study subjects into different risk groups, facilitating clinical decision-making based on risk scores. Moreover, a higher risk score was associated with a poorer prognosis for HNSC patients. The 1-year, 3-year, and 5-year AUCs of the risk scores obtained from the training and validation sets using the prognostic model were all greater than 0.6, further demonstrating the prognostic value of this model.

GSK3B plays an important role in autophagy, mainly by regulating the mTOR signaling pathway,^13^ ULK1 complex,^14^ Beclin-1 complex,^15^ TFEB,^16^ Wnt/β-catenin signaling pathway,^17^ and other pathways that regulate the initiation and progression of autophagy. And there are also have some studies shown that GSK3B promotes the proliferation and activity of tumor cells in breast cancer,^18^ prostate cancer^19^ and liver cancer.^20^ In our study, we knocked down the expression of GSK3B gene in TU686 and CAL-27 cells and we found the migration abilities of these cells descend. It can also indicate that GSK3B promotes the growth of head and neck tumor cells indirectly, but it still needs further study. Through reviewing literature, we found that the lack of GSK3B can improve obesity related metabolic disorders by promoting angiogenesis in adipose tissue.^21^ In addition, GSK3B can also affect intracellular lipid metabolism pathways by regulating various transcription factors and proteins. For example, in human hepatocellular carcinoma, GSK3B promotes cell proliferation and tumor formation by upregulating the expression of Fatty Acid Synthase (FASN);^22^ In prostate cancer, GSK3B inhibits lipid production and accumulation in tumor cells by inhibiting phosphorylation of SREBP-1.^23^ But, the specific impact and mechanism of GSK3B in head and neck tumors still need further research.

So, this study also has certain limitations. The dataset used for gene selection was relatively small, which may have reduced the support for the identified differential genes. Additionally, this study was only an analysis, and further in vitro and in vivo experiments are needed to investigate the molecular mechanisms and provide potential therapeutic targets for clinical purposes.

## Conclusion

The prognostic model constructed based on differential autophagy genes associated with head and neck tumors (CDKN2A, CSF2, GSK3B, MBTPS2, PIK3R4, SMAD2, and TRIB3) holds certain value.

## Data availability statement

The authors declare that all data are available in repository.

## Declaration of competing interest

The authors declare no conflicts of interest.
